# Abstinence from Masturbation and Hypersexuality

**DOI:** 10.1007/s10508-019-01623-8

**Published:** 2020-03-04

**Authors:** Felix Zimmer, Roland Imhoff

**Affiliations:** Social and Legal Psychology, Department of Psychology, Johannes Gutenberg University Mainz, Binger Str. 14-16, 55122 Mainz, Germany

**Keywords:** Masturbation, Abstinence, Hypersexuality, Hypersexual Behavior Inventory, DSM-5

## Abstract

Despite the lack of evidence for negative health effects of masturbation, abstinence from masturbation is frequently recommended as a strategy to improve one’s sexual self-regulation. We adopted a framework of perceived problems with pornography to collect first hints about whether abstinence from masturbation stems from a psychological and behavioral “addiction” or conflicting attitudes. In an online questionnaire survey recruited via a non-thematic Reddit thread (*n* = 1063), most participants reported that they had tried to be abstinent from masturbation. As visible from zero-order correlations and multiple linear regression, motivation for abstinence was mostly associated with attitudinal correlates, specifically the perception of masturbation as unhealthy. While there were associations with hypersexuality, no significant correlation with behavioral markers such as maximum number of orgasms was found. Higher abstinence motivation was related to a higher perceived impact of masturbation, conservatism, and religiosity and to lower trust in science. We argue that research on abstinence from masturbation can enrich the understanding of whether and how average frequencies of healthy behavior are pathologized.

## Introduction

Alongside other strategies to abstain from Internet pornography, abstinence from masturbation is advocated within a quickly growing online community. The subreddit NoFap, which currently has 516,444 followers (R/NoFap, 2019), suggests abstinence from masturbation as part of “reboot” challenges comprised of 90 days of abstinence from porn, masturbation, and orgasms. While the notion that consumption of Internet pornography is problematic has received scientific attention (Grubbs, Perry, Wilt, & Reid, 2019), abstinence from masturbation has remained unexplored. In this explorative study, we assess correlates of motivation for abstinence from masturbation in behavior and attitudes in addition to calling for abstinence from masturbation to be considered in Internet pornography research. We begin with a review of relevant developments regarding both the condemnation and acceptance of masturbation.

### Historical Perspective

Individual motivation for abstaining from masturbation has been diversely scattered across recent history. It is present in religious arguments, discussions surrounding the fear of physiological or psychological consequences, and efforts to avoid feelings of guilt or loss of control (Patton, 1986). Until the early modern age, moralists and theologians considered masturbation a “sin against nature” (Stolberg, 2000), whereas medical professionals left it largely unnoticed (Laqueur, 2003). In the beginning of the eighteenth century, this view changed with the publication of “Onania: or, the Heinous Sin of Self-Pollution,” which ascribed physiological symptoms to masturbation (Laqueur, 2003). In one edition of this work, Tissot (1781) elaborated on the concept of a “post-masturbatory disease.” He regarded the loss of semen and the mechanical manipulation of the genitals as possible causes of infection, sexual dysfunction, and insanity (Patton, 1986; Stolberg, 2000). Complementing religious arguments, fear of pathological consequences became an incentive to abstain from masturbation (Kontula & Haavio-Mannila, 2003). This fear widened to a loss of self-control or control over one’s own sexual desire (Hunt, 1998), which was linked to the ability to control and satisfy a woman and maintain the patriarchal position within the family (Stolberg, 2000). Abstinence from masturbation also reached political significance as “Victory over the sexuality of young men was symbolically necessary to provide the legitimacy for their capacity to carry forward the national or imperial project” (Hunt, 1998, p. 589). After the “masturbation panic” reached its peak in the beginning of the twentieth century (Kontula & Haavio-Mannila, 2003), progressive evaluations by medicine and psychology were on the rise (Patton, 1986). For example, Freud viewed masturbation as a natural developmental component in childhood and adolescence that should nevertheless be discarded in adulthood (Laqueur, 2003). Around the end of World War II, masturbation was regarded as a treatment option rather than a cause of psychosexual dysfunction (Patton, 1986). Finally, the “Kinsey Reports” (Kinsey, Pomeroy, & Martin, 1948; Kinsey, Pomeroy, Martin, & Gebhard, 1953) contributed to a normalized view of masturbation by revealing how widespread the behavior was across all strata of the population.

### Abstinence Motivation Today

For a period of time known as their “reboot,” the porn-critical subreddit NoFap encourages their followers to abstain from masturbation (“What is NoFap?”, 2018). They assert that “Most guys need to ‘TEMPORARILY’ [*sic*] eliminate or drastically reduce masturbation and ORGASMS [*sic*]” (Deem, 2014). In light of the recent public recommendations and the long history of masturbation panic, a scientific description and explanation of individual motivations for abstaining from masturbation are sorely needed.

The modeling of perceiving consumption of Internet pornography as problematic has already received scientific attention (Grubbs et al., 2019). Gola, Lewczuk, and Skorko (2016) studied the predictors of help-seeking behavior relevant to problematic pornography use. They reported that the quality of symptoms explains a significantly higher proportion of variance than the quantity of consumption of Internet pornography, suggesting that the frequency of use should be less diagnostically weighted to better meet the complexity of patients’ presenting concerns. Although abstinence from pornography might be regarded as a feasible intervention to alleviate any negative symptoms, no experimental investigations (but a few clinical case reports) have been made to date (Fernandez, Tee, & Fernandez, 2017). Grubbs et al. (2019) propose a two-path model comprising dysregulation and moral incongruence to explain perceived problems with pornography. Distress regarding pornography use is generated by dysregulated consumption behavior in the first pathway and by conflict with own morals or attitudes in the second. We will adopt these pathways for abstinence motivation to guide a literature review and first exploratory hypotheses.

- Pathway of physiological and psychological dysregulation. Abstinence motivation resulting from an “addiction to masturbation” characterized by a high frequency of masturbation behaviors and perceived loss of control.
- Pathway of conflicting attitudes. Abstinence motivation resulting from a “perceived addiction” characterized by conflicting attitudes that motivate reduction in an average frequency of masturbation.

To assess the aforementioned and other potential correlates, we will review contributions on masturbation frequency, hypersexuality, and selected attitudes.

### Empirical Findings

Today, learning about the pleasures of masturbation at an early age is part of European sexuality education standards (“Standards for Sexuality Education in Europe,” 2010). In a survey in the UK, about 95% of men and 71% of women reported they had masturbated at least once (Gerressu, Mercer, Graham, Wellings, & Johnson, 2008). Positive aspects of masturbation include becoming familiar with one’s own body, forming sexual fantasies, and possibly achieving sexual satisfaction without risk (Driemeyer, 2013). Furthermore, masturbation plays an important role in sex therapy (e.g., LoPiccolo & Lobitz, 1972; Zamboni & Crawford, 2003).

#### Masturbation Frequency

Despite the positive effects of masturbating, overly frequent masturbation might also have negative effects. At a purely biological level, the long reigning authoritative view is that overly frequent masturbation reduces sperm quality. This conviction encouraged the World Health Organization (2010) to recommend an intermediate duration from 2 to 7 days of sexual abstinence before sperm donation. Yet in a recent review, Ayad, van der Horst, and Du Plessis (2018, p. 245) called for a revision of this recommendation based upon finding superior sperm quality in shorter abstinence periods. On the level of physiological outcomes, thus, there is currently no evidence for any beneficial effects of abstinence from masturbation (notwithstanding endocrinological effects like an increase in serum testosterone; Exton et al., 2001; Jiang, Jiang, Zou, & Shen, 2003).

This lack of support for negative effects of frequent masturbation, however, may be markedly different for psychological variables like well-being and mental health. Two studies hint at an association of high rates of masturbation with decreased satisfaction with sexual life and life in general (Brody & Costa, 2009; Långström & Hanson, 2006). However, the authors did not control for relevant covariates such as relationship status. From a psychological perspective, extreme frequency of masturbation can be seen as a symptom of hypersexuality.

#### Hypersexuality

An early operationalization of hypersexual desire was provided by Kafka (1997), suggesting a cutoff of one orgasm per day. He also pointed out that for a subset of men, hypersexual behavior is associated with time-consuming sexual fantasies and distress. Contemporary constructs of hypersexuality include compulsive sexual behavioral disorder (Kraus et al., 2018) and hypersexual disorder (Kafka, 2010). While hypersexual disorder was rejected for the DSM-5 (Kafka, 2014), compulsive sexual behavioral disorder was included in the ICD-11. Hypersexual disorder is characterized by a long-term, frequent, and intense preoccupation with sexual fantasies and sexual behaviors that, in addition to personal suffering, leads to a reduced functioning in social, occupational, or other domains. Compulsive masturbation can be found in 30–75% of patients suffering from hypersexuality (Kaplan & Krueger, 2010). Yet, even high frequencies of masturbation must not be pathologized when they are not paired with impaired control or distress (Kraus et al., 2018).

#### Attitudes

As detailed above, masturbation abstinence cannot only be interpreted as an attempt to overcome physiological and psychological dysregulation, but also as a consequence of personal attitudes and convictions (potentially in complete absence of problematic and dysregulated behavior). Such attitude-based incentives for abstinence may be rooted in an apparent tension between religious and political core convictions and the act of masturbation, resulting in feelings of shame and guilt. Abramson and Mosher (1975) developed a measure to assess negative attitudes toward masturbation. Unsurprisingly, they found a negative correlation with the average frequency of masturbation per month, implying that persons with negative attitudes masturbate less frequently (or vice versa). They also found a high correlation of negative attitudes with sexual guilt. Sexual guilt, conceptualized as a tendency toward feelings of violation of a moral standard (Mosher, 1979), is a construct which Coleman (2003) attributed “most of the ill effects of masturbation” (p. 7) to, rather than considering the behavior itself or its frequency. Liberal sexual attitudes are connected to the general prevalence of masturbation (Das, Parish, & Laumann, 2009; Gerressu et al., 2008). Finally, religiosity has been found to be associated with negative emotions toward masturbation (Strasser, 2011) and the view of masturbation as a sin and unhealthy behavior (Davidson, Darling, & Norton, 1995).

### This Study

To our knowledge, this is the first study to exploratively assess the correlates of motivation for abstaining specifically from masturbation. Based on the pathway of physiological and psychological dysregulation, we hypothesized a positive association for hypersexuality, higher masturbation frequency before reduction, maximum number of orgasms, and earlier onset of masturbation. Representing the pathway of conflicting attitudes, we included hypotheses for religiosity, liberal attitudes, perceived impact of masturbation on everyday life, and trust in science. According to the extant findings for religiosity and liberal attitudes, we expected conservative attitudes and religiosity to be positively correlated with abstinence motivation. Furthermore, we suggest that the consideration of abstinence is often preceded by the perception that masturbation affects other areas of everyday life. Ideas about how masturbation influences concepts such as social anxiety or creativity may justify attempts to change behavior. On the contrary, limiting the relevance of masturbation to the sexual field should reduce the likelihood of considering abstinence from masturbation. Representing another possible predictor, distrust in the scientific method and scientific institutions is a topical issue in political and scholarly debates (Imhoff, Lamberty, & Klein, 2018). Since science has not provided any support for the negative view of masturbation and might even regard it as a positive and natural behavior (Robinson, Bockting, Rosser, Miner, & Coleman, 2002), trust in science was expected to be negatively related to abstinence motivation. Moreover, the conviction that masturbation poses a risk to health has been studied longitudinally in the last century (Kontula & Haavio-Mannila, 2003) and supposedly represents a strong correlate of abstinence from masturbation.

We also included sexual dysfunctions as possible correlates. For online pornography, it was investigated whether problematic consumption is related to the occurrence of erectile dysfunction. Specifically, whether a causal relationship of pornography-induced erectile dysfunction is justified (Fisher & Kohut, 2017). While there is no evidence for a general association (Landripet & Štulhofer, 2015; Prause & Pfaus, 2015), there is some evidence for problematic pornography use specifically. Yet, no causal link could be identified in a longitudinal investigation (Grubbs & Gola, 2019). Despite these findings, there is still a widespread belief in “porn induced erectile dysfunction” that motivates abstinence from pornography (Park et al., 2016) and, in case of NoFap, abstinence from masturbation as well. A reported erectile dysfunction or other sexual dysfunction might therefore be positively associated with abstinence motivation.

## Method

### Participants

Participants were recruited online via Reddit, a social news and entertainment platform. Reddit resembles an online forum with an emphasis on voting, commentary, and anonymity. It is structured by thematically specialized and autonomously moderated “subreddits.” An invitation to participate in the study was posted on several subreddits, among them also thematically relevant ones, e.g., “r/Semenretention.” The largest group of participants came from only one subreddit (“r/everymanshouldknow”), where it had been endorsed by the moderator. As this was more thematically open than most of the other subreddits where the invitation was posted and as roughly 75% of all responses (*n* = 1063) came from this subreddit, we restricted our analyses to these cases as a sample not biased by thematic fit of the subreddit. Specifically, at least one other contacted subreddit, “r/MuslimNoFap,” might have introduced severe sampling bias regarding the variable of religiosity. Conducting identical analyses with the complete sample yielded highly similar correlations. All results are available on osf.io/szhu4/.

The NoFap subreddit has also attracted women and is maintaining specific forums (Bishop, 2019). However, abstinence motivation seems to exist almost exclusively among men, as virtually all NoFap followers (99%) are male (“k31thdawson,” 2014). Inclusion criteria therefore included being over the age of 18 and a male. Two participants were excluded for speeding with a relative speed index of ≥ 2 according to Leiner (2013). Further, two participants were excluded for “straightlining,” i.e., giving the same extreme rating despite inverse coded items. Data were screened for outliers using box plots and the interquartile range, resulting in the exclusion of five datapoints lacking plausibility. The final sample included data from 1063 male participants, aged 18 years and older (*M* = 26.86, SD = 6.79). Most participants resided in North America (77.47%) and some in Europe (16.78%) or other continents (5.75%). 61.9% of the sample have acquired a university degree, while 90.69% have attended at least some college. The majority of participants (53.61%) described themselves as being in a relationship. Atheists, agnostics, and apathetics made up the overwhelming majority (70.00%) of the sample. Further, 19.80% indicated a Christian affiliation and 10.20% specified other religions. 21.6% (*n* = 225) met the criteria of hypersexuality indicated by an HBI score of ≥ 53 (Reid, Garos, & Carpenter, 2011). 23.9% (*n* = 254) suffered from at least one sexual dysfunction. Within the sample, 3.48% reported suffering from erectile dysfunction. The respective relative frequencies were 7.71% for premature ejaculation, 9.69% for difficulty orgasming, 8.84% for decreased genital sensitivity, and 4.61% for disinterest in sex.

### Measures

Included demographic variables are age, religious affiliation, and relationship status. For lack of specific hypotheses, religious affiliation was clustered into atheism (“Atheist,” “Agnostic/Apathetic”), Christian (“Christian—Protestant,” “Christian—Catholic”), and other religions (“Muslim,” “Buddhist,” “Hindu,” “Jewish,” “Mormon”). Since the variable had to be dummy-coded for the regression, the category of other religions was omitted there.

#### Abstinence Motivation

Current motivation for abstinence from masturbation (“Currently, how strong is your motivation to be/remain abstinent from masturbation?”) was captured by a slider from 0 to 100 with the poles *very weak* to *very strong*. The current mean orgasm frequency was included as a descriptor of the criterion. It was operationalized by the weekly average number of orgasms during the last 6 months.

#### Physiological and Psychological Dysregulation

Physiological and psychological dysregulation, i.e., increased preoccupation with masturbation in thoughts and behavior, was operationalized through hypersexuality, maximum number of orgasms, average masturbation frequency before reduction, and onset of masturbation and pornography consumption. The Hypersexual Behavior Inventory (Reid et al., 2011) was used to assess hypersexuality. It includes 19 items that are rated on a five-point Likert scale from *Never* to *Very Often* and comprises the subscales Coping (e.g., “I use sex to forget about the worries of daily life”), Consequence (e.g., “ I sacrifice things I really want in life in order to be sexual”), and Dyscontrol (originally named “Control,” e.g., “My attempts to change my sexual behavior fail”). The inventory exhibits a high test–retest reliability (*r* = .91) and internal consistency (*α* = .96, *α* = .94 in this sample) (Reid et al., 2011). To distinguish the different facettes of hypersexuality, the subscales were included to the analysis individually.

Although current orgasm frequency was interpreted as a correlate of abstinence (abstinent respondents have a low frequency), the masturbation history was treated as an indicator of potential “masturbation addiction.” It is conceivable that a respondent showed excessive masturbation habits (e.g., ten times per day), but is currently trying to be abstinent (presumably to deal with this excessive behavior). We thus used two items to assess behavioral markers of past sexual activity. First, participants were asked to indicate their “Total Sexual Outlets” represented by the all-time maximum number of orgasms in a week (Kinsey et al., 1948). Secondly, the average masturbation frequency per month was assessed by free indication. To achieve a parallel structure, participants who had ever been abstinent from masturbation were asked to refer to the time before the first reduction, but note that the comparability of this item between the two groups is questionable, since the average time since the first abstinence attempt was 32.5 months.

#### Conflicting Attitudes

As candidates for conflicting attitudes, we measured perceived impact of masturbation, trust in science, conservatism, religiosity, and perceived healthiness. For perceived impact of masturbation, trust in science, and conservatism, specifically developed questionnaires applying a five-point Likert scale from *strongly agree* to *strongly disagree* were used. Perceived impact was operationalized by one general item, “Masturbation behavior has an influence on other areas of everyday-life,” and ten domain-specific items. These included the perceived impact on insomnia, risk of prostate cancer, acne, creativity, productivity, tranquility, respect for a sexual partner, appreciation of physical beauty, sexual attractiveness, and emotional connection with a partner. To reduce the number of variables, we applied principal component analysis. Two components with eigenvalues 4.15 and 1.33 were extracted using parallel analysis. After varimax rotation, the variables were assigned to subscales according to their highest absolute loadings. The first subscale, termed Social Impact (*α* = .85), contained the perceived effects on productivity, tranquility, respect for a sexual partner, appreciation of physical beauty, sexual attractiveness, and emotional connection with a partner. Termed Health Impact, the second subscale (*α* = .65) was comprised of insomnia, risk of prostate cancer, acne, and creativity. The resulting subscales were correlated, *r*(1061) = .47, *p* < .001. The scale measuring Scientific Trust consisted of four items (e.g., “I value scientific knowledge higher than my own experience”) and showed only moderate internal consistency, *α* = .68. To capture conservatism, three items of the US National Survey of Family Growth (National Center for Health Statistics, 2006) were used (e.g., “A young couple should not live together unless married”). The internal consistency of the scale was acceptable, *α* = .74. Religiosity was operationalized by the annual frequency of attending church service, measured by free indication. Perceiving masturbation as unhealthy (“Overall, masturbation is”) was captured by a slider from 0 to 100 with the poles *very unhealthy* to *very healthy*. The scale was inverted so that high values represent the perception of masturbation as unhealthy.

#### Sexual Dysfunctions

Sexual dysfunctions were each measured by a dichotomous item. Participants were able to indicate whether they currently suffer from erectile dysfunction, premature ejaculation, difficulty orgasming, decreased genital sensitivity, or disinterest in sex.

### Procedure

Prior to participation, informed consent was obtained. The consent sheet as well as the questionnaire can be found online at osf.io/szhu4/. Material was presented in the order of criterion, predictors, and demographic variables. Before submission, participants were asked if they had answered all questions seriously.

To evaluate the association hypotheses, pairwise correlations and multiple linear regression were conducted. Since some variables might not be related to the criterion at all, LASSO regression (Hastie, Tibshirani, & Friedman, 2009) was used to shrink negligible predictor weights to zero and achieve a more parsimonious set of predictors which exhibits similar fit. (A full regression is included in the Appendix.) For testing null hypotheses, we adopted the threshold of *p* < .005 suggested by Benjamin et al. (2018) for claims of new discoveries.

## Results

### Descriptive Statistics

Descriptive statistics are displayed in Table 1. 64.2% (*n* = 682) of participants reported that they have tried to be abstinent from masturbation at least once. Bonferroni-corrected zero-order correlations between eligible variables are shown in Table 2. Mean orgasm frequency was not significantly correlated with abstinence motivation, *r*(1057) = − .09. A high score of hypersexuality, as measured by the overall HBI score, was correlated with greater abstinence motivation, *r*(1039) = .22, *p* < .001. Specifically, the subscales Consequence, *r*(1051) = .22, *p* < .001, and Dyscontrol, *r*(1051) = .29, *p* < .001, showed statistically relevant relationships. There were no other significant correlations representing the pathway of dysregulation. Among the potential attitudinal correlates, stronger beliefs regarding the impact of masturbation, *r*(1058) = 0.21, *p* < .001, specifically on social aspects (social impact), *r*(1060) = .20, *p* < .001, lower trust in science, *r*(1057) = − .15, *p* < .001, more conservative attitudes, *r*(1057) = .21, *p* < .001, stronger religiosity, *r*(1048) = .18, *p* < .001, and perceiving masturbation as unhealthy, *r*(932) = .41, *p* < .001, were all associated with greater motivation for masturbation abstinence. Moreover, higher prevalence of decreased genital sensitivity, *r*(1060) = .19, *p* < .001, lower age, *r*(1059) = − .15, *p* < .001, no affiliation to atheism, *r*(1059) = − .20, *p* < .001, and Christian affiliation, *r*(1059) = .14, *p* = .002, showed an association to greater abstinence motivation.

**Table 1:** Descriptive statistics

Variable	*n*	*M* (SD)	Range	Skewness
Abstinence motivation	1062	20.55 (25.20)	1, 101	1.43
Abstinence motivation^a^	1062	1.94 (1.68)	0, 4.62	0.00
Mean orgasm frequency	1060	6.69 (5.07)	0, 84	5.14
Age	1062	26.86 (6.79)	18, 71	1.66
HBI score	1042	41.91 (15.16)	19, 95	.93
HBI Coping	1054	18.10 (6.35)	7, 35	.43
HBI Consequence	1054	7.33 (3.41)	4, 20	1.38
HBI Dyscontrol	1054	16.54 (7.45)	8, 40	1.07
Max. number of orgasms	1062	16.73 (10.74)	0, 120	3.02
Masturbation frequency	1059	29.59 (33.52)	0, 651	11.03
Onset masturbation	1060	12.60 (2.00)	3, 22	− .63
Onset pornography	1057	13.55 (2.33)	4, 35	1.16
Overall impact	1061	3.16 (1.20)	1, 5	− .38
Social impact	1063	3.40 (0.86)	1, 5	− .69
Health impact	1063	3.12 (0.72)	1, 5	− .64
Trust in science	1060	3.67 (0.66)	1.50, 5	− .07
Conservatism	1060	1.72 (0.73)	1, 5	1.64
Religiosity	1051	5.58 (16.53)	0, 200	4.96
Perceiving masturbation as unhealthy	935	26.82 (23.85)	1, 101	.81

^a^Abstinence motivation was log-transformed

**Table 2:** Pairwise Pearson correlations

Variable	1	2	3	4	5	6	7	8	9	10	11	12	13
1. Abstinence motivation													
2. Mean orgasm frequency	− 0.09												
3. HBI score	0.22*	0.16*											
4. HBI Coping	0.07	0.15*	0.85*										
5. HBI Consequence	0.22*	0.17*	0.86*	0.62*									
6. HBI Dyscontrol	0.29*	0.12	0.91*	0.60*	0.77*								
7. Max. number of orgasms	− 0.11	0.53*	0.11	0.12	0.10	0.07							
8. Masturbation frequency	− 0.01	0.35*	0.11	0.10	0.12	0.10	0.35*						
9. Onset masturbation	0.09	− 0.11	− 0.02	− 0.04	− 0.03	0.00	− 0.18*	− 0.11					
10. Onset pornography	0.05	− 0.02	− 0.11	− 0.13*	− 0.05	− 0.07	− 0.00	0.04	0.40*				
11. Overall impact	0.21*	0.08	0.47*	0.37*	0.44*	0.44*	0.02	0.10	− 0.01	− 0.03			
12. Social impact	0.20*	0.02	0.35*	0.31*	0.32*	0.30*	− 0.05	0.06	0.04	− 0.03	0.53*		
13. Health impact	− 0.03	0.00	0.16*	0.23*	0.10	0.08	0.01	0.02	− 0.01	− 0.03	0.27*	0.47*	
14. Trust in science	− 0.15*	0.02	− 0.10	− 0.03	− 0.09	− 0.13	0.04	− 0.04	− 0.03	− 0.02	− 0.08	− 0.08	− 0.02
15. Conservatism	0.21*	− 0.04	0.14*	0.01	0.10	0.23*	− 0.05	− 0.01	0.10	0.16*	0.12	0.15*	0.04
16. Religiousness	0.18*	− 0.02	0.17*	0.04	0.10	0.25*	− 0.03	0.04	0.08	0.05	0.12	0.14*	0.07
17. Perceiving masturbation as unhealthy	0.41*	0.05	0.44*	0.22*	0.40*	0.52*	− 0.03	0.05	0.08	− 0.02	0.31*	0.21*	− 0.03
18. Erectile dysfunction	0.11	0.03	0.11	0.05	0.12	0.14*	− 0.01	0.00	− 0.01	− 0.06	0.06	0.05	− 0.00
19. Premature ejaculation	0.05	− 0.03	0.11	0.04	0.13	0.14*	0.05	− 0.00	− 0.02	− 0.04	0.06	0.05	− 0.00
20. Difficulty orgasming	0.08	0.06	0.21*	0.15*	0.18*	0.20*	− 0.01	0.02	0.01	− 0.04	0.10	0.07	0.03
21. Decreased genital sensitivity	0.19*	0.04	0.24*	0.13	0.21*	0.26*	− 0.01	0.02	0.02	− 0.07	0.10	0.11	− 0.01
22. Disinterest in sex	0.08	− 0.04	0.15*	0.09	0.10	0.16*	− 0.02	− 0.01	0.03	− 0.04	0.09	0.07	0.02
23. Age	− 0.15*	0.00	− 0.06	− 0.03	− 0.02	− 0.09	0.14*	0.03	− 0.07	0.18*	− 0.10	− 0.11	− 0.02
24. Relationship status	− 0.12	− 0.03	− 0.14*	− 0.07	− 0.10	− 0.17*	0.06	0.00	− 0.09	− 0.02	− 0.08	− 0.04	0.06
25. Atheism	− 0.20*	0.00	− 0.17*	− 0.07	− 0.12	− 0.22*	− 0.03	− 0.01	− 0.09	− 0.07	− 0.10	− 0.08	− 0.02
26. Christian	0.14*	− 0.00	0.09	0.04	0.03	0.14*	0.00	− 0.00	0.07	0.07	0.08	0.08	0.02
27. Other religious affiliation	0.13	0.02	0.17*	0.09	0.17*	0.20*	0.02	0.01	0.03	0.02	0.07	0.08	0.00

Variable	14	15	16	17	18	19	20	21	22	23	24	25	26
14. Trust in science													
15. Conservatism	− 0.24*												
16. Religiousness	− 0.08	0.54*											
17. Perceiving masturbation as unhealthy	− 0.18*	0.34*	0.32*										
18. Erectile dysfunction	− 0.02	0.02	− 0.02	0.09									
19. Premature ejaculation	− 0.03	− 0.02	− 0.00	0.12	0.14*								
20. Difficulty orgasming	0.01	− 0.01	0.01	0.10	0.18*	− 0.04							
21. Decreased genital sensitivity	− 0.01	0.05	0.08	0.17*	0.25*	0.02	0.33*						
22. Disinterest in sex	− 0.01	0.05	0.03	0.14*	0.13	0.02	0.25*	0.22*					
23. Age	− 0.01	0.07	− 0.07	− 0.21*	0.04	0.03	0.00	− 0.03	− 0.02				
24. Relationship status	0.03	− 0.02	− 0.03	− 0.23*	− 0.05	0.00	− 0.06	− 0.05	− 0.08	0.32*			
25. Atheism	0.23*	− 0.43*	− 0.43*	− 0.22*	− 0.09	0.01	− 0.03	− 0.08	− 0.04	0.03	0.03		
26. Christian	− 0.15*	0.37*	0.35*	0.14	0.07	− 0.05	− 0.01	0.04	0.03	− 0.02	0.01	− 0.76*	
27. Other religious affiliation	− 0.09	0.18*	0.22*	0.19*	0.07	0.08	0.08	0.11	0.03	0.01	− 0.08	− 0.36*	− 0.12

Abstinence motivation was log-transformed. Bonferroni correction was applied on *p* values for *N *= 351 comparisons

*p < .005

### Regression

Before conducting the regression and LASSO predictor selection, statistical assumptions were checked. The homoscedasticity of residuals was violated. This was addressed via Box-Cox transformation of the criterion (Sakia, 1992), specifically a logarithm transformation. Normality of residuals could not be met after correction. However, since sample size is considered large (the number of observations per variable is > 10), a marked impact on bias and tests is unlikely (Schmidt & Finan, 2018).

Tests for multicollinearity indicated that a low level of multicollinearity was present (VIF_max_= 3.37 in the full model, VIF_max_ = 2.59 in the LASSO reduced model). Results of the regression predicting motivation for abstinence from masturbation are displayed in Table 3. The overall proportion of explained variance was moderate, *R*_adj_^2^ = .205, *F*(17, 829) = 13.84, *p* < .001. Motivation for abstinence from masturbation was significantly predicted by the perceived social impact of masturbation and the perception of masturbation as unhealthy. HBI Dyscontrol, HBI Coping, and decreased genital sensitivity also contributed variance explanation on trend level, *p* < .05. In contrast to expectations, using sexual activity for coping purposes implied lower abstinence motivation. Other variables contributed to the overall prediction according to the LASSO procedure, but did not possess significant predictor weights.

**Table 3:** Predictors of abstinence motivation

Predictor	*β*	99.5% CI	*t*	*p*
HBI Coping	− 0.10	− 0.21, 0.01	− 2.45	.014
HBI Dyscontrol	0.10	− 0.03, 0.23	2.10	.036
Max. number of orgasms	− 0.06	− 0.16, 0.03	− 1.78	.075
Masturbation frequency	− 0.06	− 0.16, 0.03	− 1.82	.070
Onset pornography	0.04	− 0.05, 0.13	1.26	.209
Overall impact	0.04	− 0.07, 0.14	0.93	.355
Social impact	0.12	0.01, 0.24	2.99	.003
Health impact	− 0.07	− 0.17, 0.04	− 1.84	.066
Trust in science	− 0.06	− 0.15, 0.03	− 1.88	.060
Perceiving masturbation as unhealthy	0.27	0.16, 0.38	7.01	<.001
Erectile dysfunction	0.02	− 0.07, 0.11	0.69	.491
Difficulty orgasming	0.03	− 0.06, 0.13	1.01	.311
Decreased genital sensitivity	0.08	− 0.01, 0.18	2.40	.016
Disinterest in sex	− 0.04	− 0.13, 0.05	− 1.16	.245
Age	− 0.06	− 0.15, 0.03	− 1.94	.053
Atheism	− 0.07	− 0.21, 0.06	− 1.51	.132
Christian	0.01	− 0.12, 0.15	0.24	.808

Predictor selection has been performed via LASSO shrinkage. Excluded predictors were: HBI Consequence, onset masturbation, health impact, conservatism, religiosity, premature ejaculation, relationship status. *n* = 847, *R*_adj_^2^ = .205

## Discussion

This explorative study aimed to evaluate the associations of motivation for abstinence from masturbation. On the level of zero-order correlations and multiple linear regression, support for both hypothesized pathways, physiological and psychological dysregulation, and conflicting attitudes, was found. Yet, evidence for a pathway of conflicting attitudes was richer in quantity and quality.

For the pathway of physiological and psychological dysregulation, which can be conceptualized as a “masturbation addiction,” only the subscales of the HBI were associated with abstinence motivation. The HBI subscale Consequence as well as Dyscontrol showed positive associations to abstinence motivation, yet only Dyscontrol showed variance explanation within the regression model. Since abstinence from masturbation is an endeavor of controlling sexual behavior, the connection to feelings of dyscontrol regarding sexual activity is unsurprising. For the HBI subscale Coping, there was no zero-order correlation, but a significant negative relationship with the regression criterion was found. This implies that higher ratings on items such as “I use sex to forget about the worries of daily life” have been accompanied by less motivation to abstain. A possible explanation is that a functional role of masturbation, e.g., as a coping mechanism, for relaxation, etc., is a motivational counterpart to efforts to abstain. Other variables assigned to this pathway, the mean masturbation frequency before reduction, maximum number of orgasms, and onsets of masturbation and pornography consumption, showed no significant zero-order correlation or variance explanation in the regression. Descriptively, the all-time maximum number of orgasms was even lower in men with high abstinence motivation and vice versa, *r*(845) = − 0.11, *p* = .001 (without Bonferroni correction). Although it cannot be taken as a proof of the null, it speaks toward a low relevance of behavioral variables in the phenomenon of abstinence motivation.

The other pathway explains abstinence motivation by conflicting attitudes, specifically higher perceived impact, lower trust in science, higher conservatism, religiosity, and belief in a negative health impact. In zero-order correlations, all of these associations except for one subscale of perceived impact could be confirmed in the hypothesized direction. In the regression model, only social impact and perception of masturbation as unhealthy achieved significant variance explanation while exhibiting the largest predictor weights. Interestingly, the associations with the two facets of the perceived impact, health and social, pointed in different directions. Contrary to expectation, perceived impact of masturbation on health-related variables (e.g., cancer or acne) showed no zero-order correlation and even tended toward a negative predictor weight in the regression (*β* = − .07, *p* = .066). These results suggest that seeing a possibility to improve social life, rather than to avoid illnesses, might promote abstinence motivation. Summarizing the evidence from both pathways, abstinence motivation was mostly associated with attitudinal correlates, specifically the perception of masturbation as unhealthy.

Due to ongoing debates about pornography-induced sexual dysfunctions, we considered them as potential correlates of abstinence motivation. Of the five candidates, only men suffering from decreased genital sensitivity showed a higher abstinence motivation. Rather than viewing masturbation as problematic, one suggested line of interpretation is a reduced incentive to masturbate.

### Limitations and Future Research

The main limitation of this study is the exploratory nature and the loose attachment to a theoretical framework. Specifically, the usage of the pathway model on another level of analysis, namely motivation for abstinence instead of the originally applied problem awareness, and post hoc assignment of the variables to the two paths, shall be discussed. To seamlessly transfer the model, one needs to assume an obvious theoretical step from problem awareness to abstinence motivation. Yet, there are other plausible pathways leading to abstinence motivation. For example, it can also be part of an effort to change sexual outlet toward more penile–vaginal intercourse. The interpretation of the association with decreased genital sensitivity also applied the possibility of abstinence motivation without the view of masturbation behavior as problematic. Therefore, it remains debatable whether the pathway model is suitable for abstinence motivation. Secondly, the assignment of the studied variables to the pathways of dysregulation and conflicting attitudes is not unambiguous for all variables. Take the HBI item “I do things sexually that are against my values and beliefs” for example. In this study, it was assigned to the pathway of dysregulation for its function as a marker of hypersexuality. However, it fits in perfectly with the pathway of attitudinal correlates, since an arbitrary amount of sexual activity, determined solely by moral standards, can justify a high score for the item.

The majority of participants of this study presumably were visitors or subscribers of the subreddit “r/everymanshouldknow.” Although limiting analyses to this subsample were performed in an attempt to reduce sampling bias (see Methods section), it remains questionable whether conclusions can be extrapolated to an intended population of male adults. On a theoretical note, sampling bias might be introduced by correlates of the apparent affinity toward a *manliness* theme such as more conservative sexual attitudes and behavior. Empirically, the average HBI sum score of 41.91 (SD = 15.16) showed a significant deviation from a previous “healthy” sample (*M* = 34.2, SD = 14.5, *n* = 147, Reid et al., 2011, *t*(1187) = 5.80, *p* < .001) indicating a relatively increased prevalence of hypersexuality within this sample. Therefore, it cannot be ruled out that associations with masturbation abstinence differ in the general population. Another important limitation is the cross-sectional nature of the study and the associated limitations regarding causal inferences. For example, since the HBI is a self-administered tool and open to subjective interpretation (e.g., “My sexual behavior controls my life”), the causal direction of an association between an HBI score and abstinence motivation remains unclear. According to the pathway of conflicting attitudes, pathologization of average frequencies of behavior might also lead to notions of excessive behavior and high HBI scores.

The scope for study design improvements is particularly evident in the variables covered. Asking about current abstinence from masturbation and the view of one’s own masturbation as problematic should be included in future research. This would also facilitate comparison to existing research on Internet pornography. Furthermore, abstinence motivation might not be a criterion of preference. Within this study, 36.3% of the participants reported no motivation on a scale from 0 to 100, which due to the need for transformation can be considered a high limitation of variance. Assuming a normal distribution of the underlying construct, an item with higher difficulty, e.g., Do you consider reducing your frequency of masturbation?” may resolve this issue. 64.2% of participants in this study indicated that they have tried to be abstinent from masturbation at least once. Although we could not find a comparative figure, we regard it as unexpectedly high and possibly subject to scrutiny. Regarding sexual dysfunction, our questionnaire design prevented us from differentiating the indication of no sexual dysfunctions and otherwise missing values, e.g., lacking willingness of specification.

Although these limitations represent notable reservations, we would like to emphasize that the focus of this study is to encourage further efforts to design and eventually test hypotheses. It has already been demonstrated that inclusion of masturbation can be fruitful for understanding correlates of pornography consumption. For example, solo masturbation might actually explain the negative association of pornography viewing and relationship quality (Perry, 2019). Understanding the constituents of both abstinence from pornography and abstinence from masturbation might eventually be a basis for reducing pathologization of average and healthy frequencies of sexual behavior.

## Appendix

See Table 4.

**Table 4:** Predictors of abstinence motivation

Predictor	*β*	99.5% CI	*t*	*p*
HBI Coping	− 0.10	− 0.22, 0.02	− 2.38	.018
HBI Consequence	− 0.00	− 0.15, 0.14	− 0.07	.942
HBI Dyscontrol	0.11	− 0.05, 0.27	1.89	.058
Max. number of orgasms	− 0.06	− 0.16, 0.03	− 1.86	.064
Masturbation frequency	− 0.06	− 0.16, 0.03	− 1.87	.062
Onset masturbation	− 0.02	− 0.12, 0.08	− 0.62	.536
Onset pornography	0.05	− 0.05, 0.15	1.45	.149
Overall impact	0.03	− 0.07, 0.14	0.88	.378
Social impact	0.12	0.01, 0.24	3.02	.003
Health impact	− 0.07	− 0.17, 0.04	− 1.79	.073
Trust in science	− 0.06	− 0.15, 0.03	− 1.87	.062
Conservatism	− 0.02	− 0.13, 0.10	− 0.42	.675
Religiosity	− 0.01	− 0.12, 0.10	− 0.33	.743
Perceiving masturbation as unhealthy	0.28	0.17, 0.40	6.92	<.001
Erectile dysfunction	0.02	− 0.07, 0.11	0.66	.508
Premature ejaculation	− 0.01	− 0.10, 0.08	− 0.27	.790
Difficulty orgasming	0.03	− 0.06, 0.13	0.96	.338
Decreased genital sensitivity	0.08	− 0.01, 0.18	2.39	.017
Disinterest in sex	− 0.04	− 0.13, 0.05	− 1.14	.254
Age	− 0.06	− 0.16, 0.03	− 1.83	.068
Relationship status	0.01	− 0.09, 0.10	0.21	.832
Atheism	− 0.08	− 0.23, 0.06	− 1.64	.102
Christian	0.01	− 0.12, 0.15	0.26	.793

*n* = 847, *R*_adj_^2^ = .200
